# Seeing the truck, but missing the cyclist: effects of blur on duration thresholds for road hazard detection

**DOI:** 10.1186/s41235-024-00557-7

**Published:** 2024-05-20

**Authors:** Silvia Guidi, Anna Kosovicheva, Benjamin Wolfe

**Affiliations:** Department of Psychology, University of Toronto Mississauga, 3359 Mississauga Road, Mississauga, ON L5L 1C6 Canada

**Keywords:** Driving, Scene perception, Blur, Hazard detection

## Abstract

Drivers must respond promptly to a wide range of possible road hazards, from trucks veering into their lane to pedestrians stepping onto the road. While drivers’ vision is tested at the point of licensure, visual function can degrade, and drivers may not notice how these changes impact their ability to notice and respond to events in the world in a timely fashion. To safely examine the potential consequences of visual degradation on hazard detection, we performed two experiments examining the impact of simulated optical blur on participants’ viewing duration thresholds in a hazard detection task, as a proxy for eyes-on-road duration behind the wheel. Examining this question with older and younger participants, across two experiments, we found an overall increase in viewing duration thresholds under blurred conditions, such that younger and older adults were similarly impacted by blur. Critically, in both groups, we found that the increment in thresholds produced by blur was larger for non-vehicular road hazards (pedestrians, cyclists and animals) compared to vehicular road hazards (cars, trucks and buses). This work suggests that blur poses a particular problem for drivers detecting non-vehicular road users, a population considerably more vulnerable in a collision than vehicular road users. These results also highlight the importance of taking into account the type of hazard when considering the impacts of blur on road hazard detection.

**Supplementary Information:**

The online version contains supplementary material available at 10.1186/s41235-024-00557-7.

## Significance statement

While visual impairment has well-established impacts on driver behaviour, understanding the specific impacts it has on different road users is essential. Most roads are shared by passenger and commercial vehicles, non-vehicular road users (pedestrians and cyclists), and, unintentionally, by animals. In a hazardous situation, it is imperative that drivers detect other road users in a timely manner in order to avoid a collision, and non-vehicular road users are uniquely vulnerable. Our results here suggest that non-vehicular road users are specifically impacted by visual degradation in the form of blur, suggesting that focused interventions may pay substantial dividends in improving road safety for all road users. Moreover, our results suggest that blur impacts the process of avoiding a collision at the level of perceptual judgments, indicating a need to focus on the perceptual stage of collision avoidance to ensure safety for all road users.

## Introduction

Driving is a visual task in which we must attend to our surroundings to perceive and react to a range of hazardous situations. Without proper visual function, a driver’s ability to notice and safely respond to hazards is significantly impaired. Visual impairments such as glaucoma, age-related macular degeneration, and cataracts make it harder for drivers to respond to hazards (Haymes et al., 2008; Wood et al., 2018), increasing the time they need to do so (Wood et al., 2021), and increasing the chance of a collision (Owsley et al., 1998; Wood & Black, 2016). Even in the absence of pathology, driving performance can be impaired by uncorrected refractive error (Wood et al., 2014), or normal age-related losses in visual function (Ortiz-Peregrina et al., 2020; Wood, 2002). Furthermore, since vision losses are often gradual, they can escape notice until their impact is severe (Carberry et al., 2006; Johnson & Keltner, 1983).

Given the real-world impact of visual impairment on driving performance, previous work has examined the effects of visual degradation, in the form of blur, on road hazard detection (Lacherez et al., 2019; Lee et al., 2016; Swan et al., 2019; Wood et al., 2010, 2014). These range from computer-based video studies to closed-track driving and simulator studies in which optical blur is added using lenses in otherwise normally-sighted observers. For example, on-road, closed-track studies have shown that reduced acuity impaired older and younger drivers’ ability to drive safely (Wood et al., 2009), their sign recognition ability (Wood et al., 2014), and increased the likelihood of collisions (Wood et al., 2014).

Similar results have been shown in studies wherein participants view dashboard camera videos, which have the advantage of closely matching real hazards in terms of their visual characteristics, variability, and complexity. The Hazard Perception Test (HPT) has been widely used to probe drivers’ understanding of road scenes, requiring participants to respond to videos recorded from the drivers’ perspective that involve a range of hazardous situations (e.g., pedestrians, other vehicles). Lee and colleagues (2016) used the HPT to determine how long drivers took to indicate where a hazard was in a video of a road scene viewed in a laboratory setting. Blur increased participants’ reaction times to hazards, and increased fixation durations on hazards prior to their response.

While these results highlight the detrimental effects of blur on road hazard perception, they may not affect all road users equally. Since visual function declines with age, a key question is whether blur impacts older drivers differently than younger drivers. In a closed-track driving study, Wood et al. (2009) showed that blur affected older drivers’ performance as measured by road sign recognition and time to complete the course. In a laboratory study using the HPT, Lee et al. (2016) showed that the impact of blur was similar for older and younger participants, increasing reaction times to hazards. However, previous work in other domains has shown evidence that older adults may be *more* resistant to blur when reading text signs (Kline et al., 1999) and while identifying driving-relevant features in photographs of road scenes (Lee et al., 2015). Some of these effects may be task- and stimulus dependent, as other work has shown older adults may be less resistant to the effects of blur in other settings (e.g., Bartel & Kline, 2002; Wolfe et al., 2016).

Another consideration in examining the differential impacts of blur for older and younger drivers is the effect that it may have on different aspects of the decision process. Drivers must determine whether there is a hazard, and then plan and implement an appropriate response. Measures commonly used in driving studies, like reaction time or detection rates, have significant practical relevance, but reflect a combination of perceptual (e.g., object recognition) and post-perceptual processes (e.g., planning, response preparation). However, when comparing older and younger drivers, motor and decisional processes are likely to differ, making reaction times challenging to interpret. Moreover, understanding the effects of blur at different stages of the process is important for understanding the extent to which they are amenable to different interventions. Increments in reaction time produced at the decisional level, for example, may require different interventions (e.g., driver training) than vision-based processes. This can be addressed by isolating one stage of the process (e.g., hazard detection), by measuring duration thresholds for road hazard detection—i.e., the minimum time required to *detect* a hazard. This measure is analogous to eyes-on-road time, the time that drivers spend looking at the forward roadway while performing driving related tasks in in-vehicle or simulator studies.

Furthermore, blur may differentially impact detection of specific hazards. Previous studies have largely focused on specific subsets of hazards (e.g., pedestrians) or measured reaction times to hazards as a broad category within hazard perception tests, which include vehicular and non-vehicular hazards together. However, the distinction between vehicular and non-vehicular hazards is important in the context of road safety planning and transportation research, as non-vehicular road users (e.g., pedestrians, cyclists) are at greater risk of injury in collisions and are commonly classified as vulnerable road users.

Hazards on the road vary widely in size, and blur, which removes fine detail, may impact some classes of hazards. A driver might be able to notice a truck simply because it is large, whereas a pedestrian, more defined by high spatial frequencies, might be harder to see. Not all hazards are equal, and while this kind of selective loss of information has been studied in road sign identification (Kline et al., 1999) it has not been applied here.

In two experiments, we examined how blur impacts the viewing duration required to detect road hazards and whether this varies across age groups. We used the Road Hazard Stimuli (Song et al., 2023; Wolfe et al., 2020), a corpus of dashcam videos of dangerous road situations. Rather than measuring reaction time, as done in the HPT, we measured viewing duration thresholds, isolating the perceptual processes underlying hazard detection. As this is the first study to assess the impact of blur on hazard detection using duration thresholds, we ran an initial experiment to examine the impact of blur on thresholds using a mixed set of vehicular and non-vehicular hazards. We hypothesized that blur would increase duration thresholds, reflecting impairments in object identification, and that thresholds would be higher for older participants. We then conducted a second experiment, to extended this paradigm, adding an intermediate level of blur and categorizing hazards as vehicular and non-vehicular to determine whether blur had differential effects by hazard type. We predicted that blur would selectively increase duration thresholds for non-vehicular compared to vehicular hazards, and that smaller levels of blur would be sufficient to impact duration thresholds for nonvehicular hazards.

## Methods

### Participants

In Experiment 1, 86 participants completed the study online through Prolific. Six participants’ data were removed based on preregistered exclusion criteria (see Analysis), for a final sample of 80. All observers had self-reported normal or corrected-to-normal vision, a valid drivers’ license, and were a resident of Canada, the United States, or the United Kingdom. The final sample consisted of 40 younger (ages 20–35; mean: 26.1) and 40 older participants (ages 55–69; mean: 60.6). Experiment 2 used the same criteria, with a higher age range for older adults. We recruited 104 participants (24 were removed; see Analysis) for a final sample of 80 new participants, 40 younger (ages 20–35, mean: 20.9 years) and 40 older (ages 60–74; mean: 65.7 years). All participants provided informed consent prior to participating.

### Stimuli

Videos were drawn from the Road Hazard Stimuli, a set of naturalistic videos containing hazardous road situations filmed from forward-facing dashcams, with a matched set of non-hazard videos (for details, see Song et al., 2023; Wolfe et al., 2020). Hazards varied widely, including pedestrians and vehicles in a range of environments (e.g., highway, city streets), weather, and lighting conditions. For this study, hazards were defined as situations requiring an immediate driver response (e.g., braking, swerving), and videos had been previously annotated for the onset time of the hazard and the onset time of the driver response (for details, see Wolfe et al., 2020). Matched non-hazard videos were clips taken from the hazard-present videos from time stamps at least 10 s before or after hazard onset, when available. Therefore, the video environment was the same, but no hazards were present.

In Experiment 1, videos were drawn from a possible set of 195 hazard-present videos (consisting of a mix of 158 vehicular and 37 non-vehicular hazards) and 251 hazard-absent videos; these videos were drawn from Wolfe et al. (2020). In Experiment 2, to gather a sufficient number of vehicular and non-vehicular hazard videos, this stimulus set was expanded to include a possible 311 hazard-present videos (keeping the same 251 hazard absent videos); these videos represent a subset of those reported in Song et al. (2023) and consist of 197 vehicular and 114 non-vehicular hazards. All videos had a resolution of 1280 × 720 pixels and a frame rate of 30 frames per second. For the blur conditions, videos were computationally blurred using a circular kernel. The high blur condition, used in Experiments 1 and 2, used a kernel diameter of 10.8 pixels, corresponding to a high-frequency cutoff (half-max) of 47 cycles/image. The low blur condition, used only in Experiment 2, used a kernel diameter of 5.4 pixels (cutoff of 94 cycles/image). At an approximate viewing distance of 40 cm and video height of 14 cm (36 pixels/deg), these values correspond to 1.7 and 0.9 diopters of blur, respectively, as seen by the participant (calculated from Strasburger et al., 2018). These values were selected from a combination of piloting and blur levels used in previous studies that used optical blur (e.g., Lee et al., 2016; Wood et al., 2014). In Experiment 2, thresholds were separately estimated for videos containing vehicular hazards (e.g., cars, SUVs, trucks, buses) and non-vehicular hazards (pedestrians, cyclists, animals).

### Procedure

**Fig. 1:**
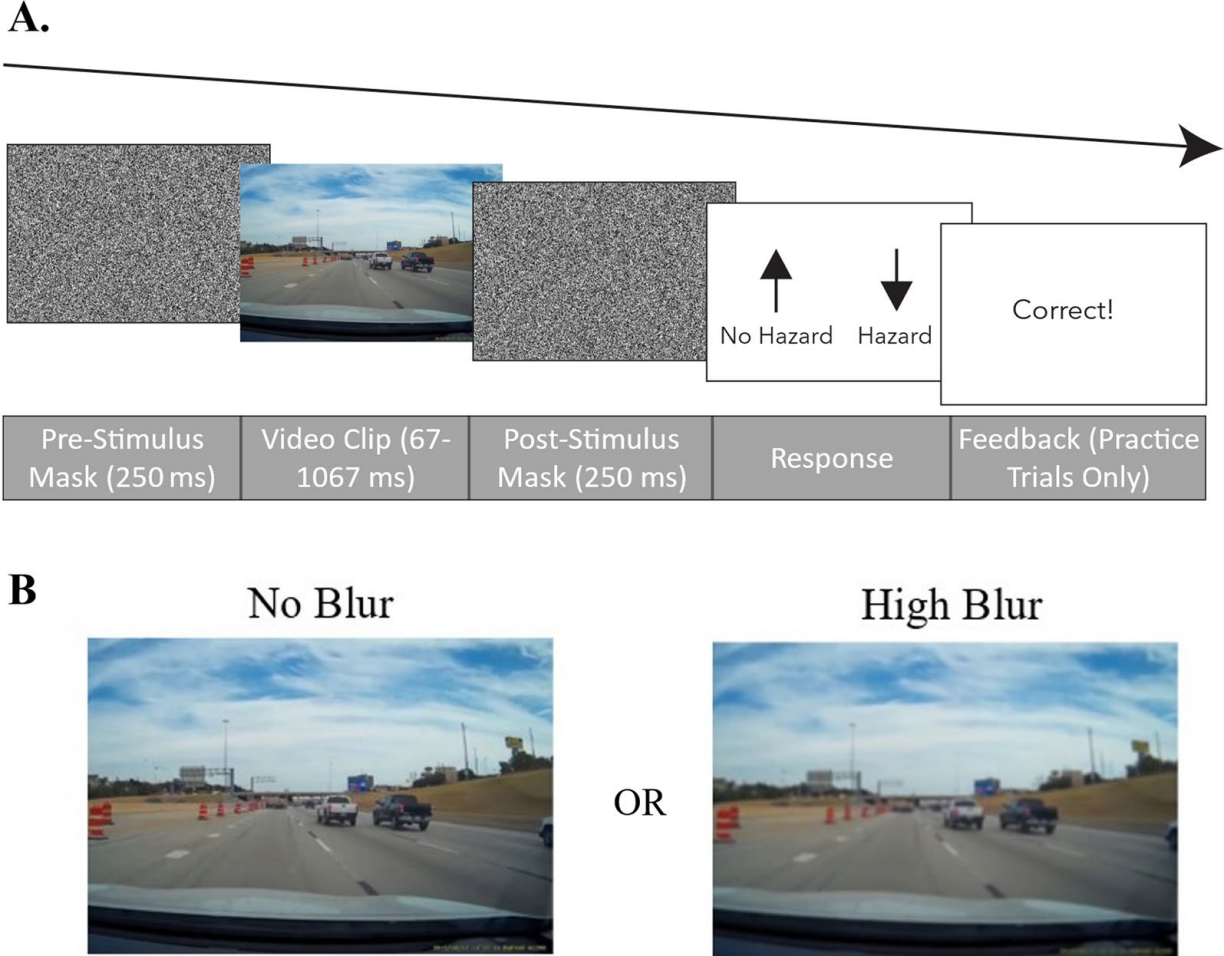
Experiment 1 set-up and trial sequence. **A** Each trial began with a noise mask shown for 250 ms, followed by a blurred or non-blurred video (67–1067 ms), followed by a noise mask. The participants’ task was to report whether a hazard was present in the clip. Feedback was only provided in practice trials. **B** Illustration of the no blur (left) and high blur (right) conditions

Both experiments were developed in PsychoPy/PsychoJS (v. 2020) (Peirce et al., 2019) and hosted on Pavlovia. At the time of the study, PsychoPy/PsychoJS was the lowest-latency platform for online behavioral studies (Bridges et al., 2020). To verify the accuracy of stimulus timing and video playback, we conducted a separate experiment to replicate previous in-lab results (Wolfe et al., 2020; see Supplemental Materials). Participants completed the study on a desktop or laptop computer, and trials followed the sequence shown in Fig. 1. Each trial began with a pre-stimulus mask (250 ms), followed by a video clip (lasting between 67 and 1067 ms), followed by a post-stimulus mask and response screen. Participants’ task was to report whether or not they saw a hazard by pressing one of two arrow keys (Fig. 1).

For hazard-present videos, video segments were taken from the interval immediately preceding the time of the annotated driver response. This was done to prevent participants from relying on cues related to the driver’s response to make their judgments (e.g., changes in optic flow from the driver suddenly braking or swerving). Each video ended at the time of the annotated driver response. The start time of each video was determined by subtracting the video duration on that trial (based on the staircase) from the time point of the annotated driver response. All video content shown was between the onset of the hazard (first visible deviation of the hazard from its normal state) and the time of the driver response (see Wolfe et al., 2020, for details). Blur conditions were always randomly interleaved, and hazard prevalence was 50% (i.e., half the trials contained a hazard, and half did not). The blurred videos that were presented to each participant were randomly chosen from the stimulus set such that any given video for a participant was randomly assigned to a given blur condition. Videos were not repeated within participants, and were randomly drawn from the stimulus set. Given the numbers of the videos in the stimulus set relative to the number of trials, each participant saw most of the videos, but not every video was seen by every participant.

Video durations for each blur condition in Experiments 1 and 2 were independently controlled by separate, interleaved adaptive staircases, with either 100 or 72 trials per condition (Experiments 1 and 2, respectively). The duration of the first video within each staircase was set to 600 ms and then changed based on performance using a modified three-down, one-up rule. The initial step size was 133 ms and decreased to 67 ms after six reversals. To increase efficiency, the staircase followed a 1-up, 1-down rule until the first reversal. Experiment 2 adapted this procedure, reducing the trial count to 72 trials per condition to enable the use of six separate staircases (3 blur conditions [no, low, high] × 2 hazard types [vehicular, non-vehicular]) without repeating videos. Experiment 2 used the same procedure as Experiment 1.

Each experiment included randomly interleaved catch trials (20 in Experiment 1, 36 in Experiment 2) wherein participants were shown a 1 s clip of a non-driving scene, and asked to report whether it had been an indoor or an outdoor scene. Clips used for the catch trials were sourced from a stock footage website (Pexels.com; under a free use license) and depicted a variety of everyday settings (e.g., beach, garden, forest, kitchen, library) and activities (e.g., hiking, cleaning, cooking, running). Including the catch trials, participants completed 220 experimental trials in Exp 1, and 468 in Experiment 2. Participants also completed a 40 trial practice session with feedback prior to beginning the experiment, but no feedback was provided during the experiment.

### Analysis

Duration thresholds were estimated by fitting psychometric functions to each participant’s data in each condition. Based on preregistered exclusion criteria, we excluded participants with unreliable psychometric function fits, based on extreme duration thresholds (outside the range of 0–1500 ms; 2 and 9 participants removed in Experiments 1 and 2, respectively), or an increment of less than 5% accuracy per second of video in one or more conditions, determined from a linear fit to the data (1 and 12 participants in Experiments 1 and 2). The larger number of participants excluded in Experiment 2 is likely due to the more stringent requirement to have six (rather than two) reliable psychometric fits, each of which was based on fewer trials (72 instead of 100). We also re-analyzed the data by determining the threshold using the average of all the reversals in each staircase, which produced similar results (see Supplemental Materials).

In addition, participants needed to achieve higher-than-chance levels of accuracy on the catch trials, based on a binomial test, to be included in the analysis (no participants were excluded based on this). Mean accuracy on catch trials was 98.2% (SD: 3.6%) and 98.4% (SD: 2.0%) in Experiments 1 and 2, respectively. Finally, we excluded participants for whom their reported age did not match the age shown on their Prolific profile (3 participants each in Exp 1 and 2).^1^

For each condition and participant, response accuracy (based on both hazard present and hazard absent trials) was fit with a two-parameter cumulative Gaussian function (mean and standard deviation) using a maximum likelihood criterion, with floor and ceiling constrained at 50% and 100%, respectively, and thresholds were defined as the duration required to achieve 80% accuracy. In Experiment 1, duration thresholds were compared using a 2 (blur conditions; within-subjects) ⨯ 2 (age group; between subjects) mixed-model ANOVA. In Experiment 2, six duration thresholds were calculated for each participant, and they were compared using a 3 (Blur Level: No, Low, High) ⨯ 2 (Hazard Type: Vehicular vs. Non-vehicular) ⨯ 2 (Age Group: Younger and Older) mixed-model ANOVA. We report post-hoc comparisons using the Tukey method for multiple comparisons, with adjusted p-values (compared against α = 0.05).

## Results

**Fig. 2:**
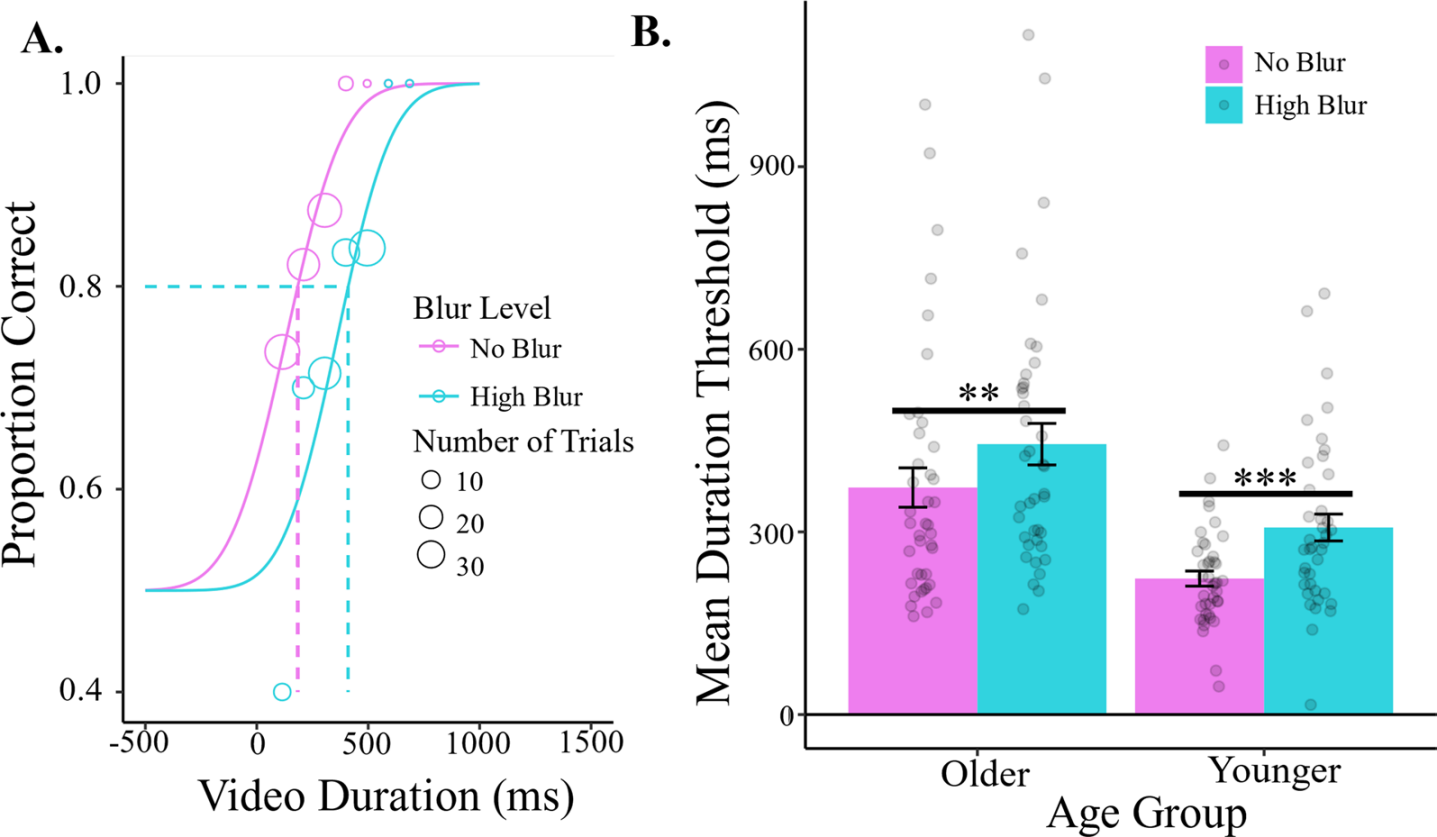
Results for Experiment 1. **A** Accuracy as a function of video duration from one representative participant, showing data from the no blur (magenta) and blur (cyan) conditions. Dotted lines indicate 80% duration thresholds. Circle size corresponds to the number of trials at that duration. **B** Group mean plots for duration threshold, separated by age group and blur (magenta, no blur; cyan; blur) condition. Error bars are ± 1 standard error of the mean and each point represents one participant’s threshold. (** = *p* < 0.01. *** = *p* < 0.001)

In Experiment 1, we observed higher duration thresholds for older adults (*M* ± *SD*; 409 ± 212 ms) compared to younger adults (265 ± 120 ms), *F*(1,78) = 19.34, *p* < 0.001, *η*_*p*_^2^ = 0.20. Blur significantly impacted duration thresholds, *F*(1,78) = 16.65, *p* < 0.001, *η*_*p*_^2^ = 0.18; higher thresholds with blur (376 ± 193 ms) vs no blur (298 ± 171 ms). The effects of blur were comparable between the age groups, with no significant interaction, *F*(1,78) = 0.11, *p* = 0.74,, *η*_*p*_^2^ = 0.001 (Fig. 2).

**Fig. 3:**
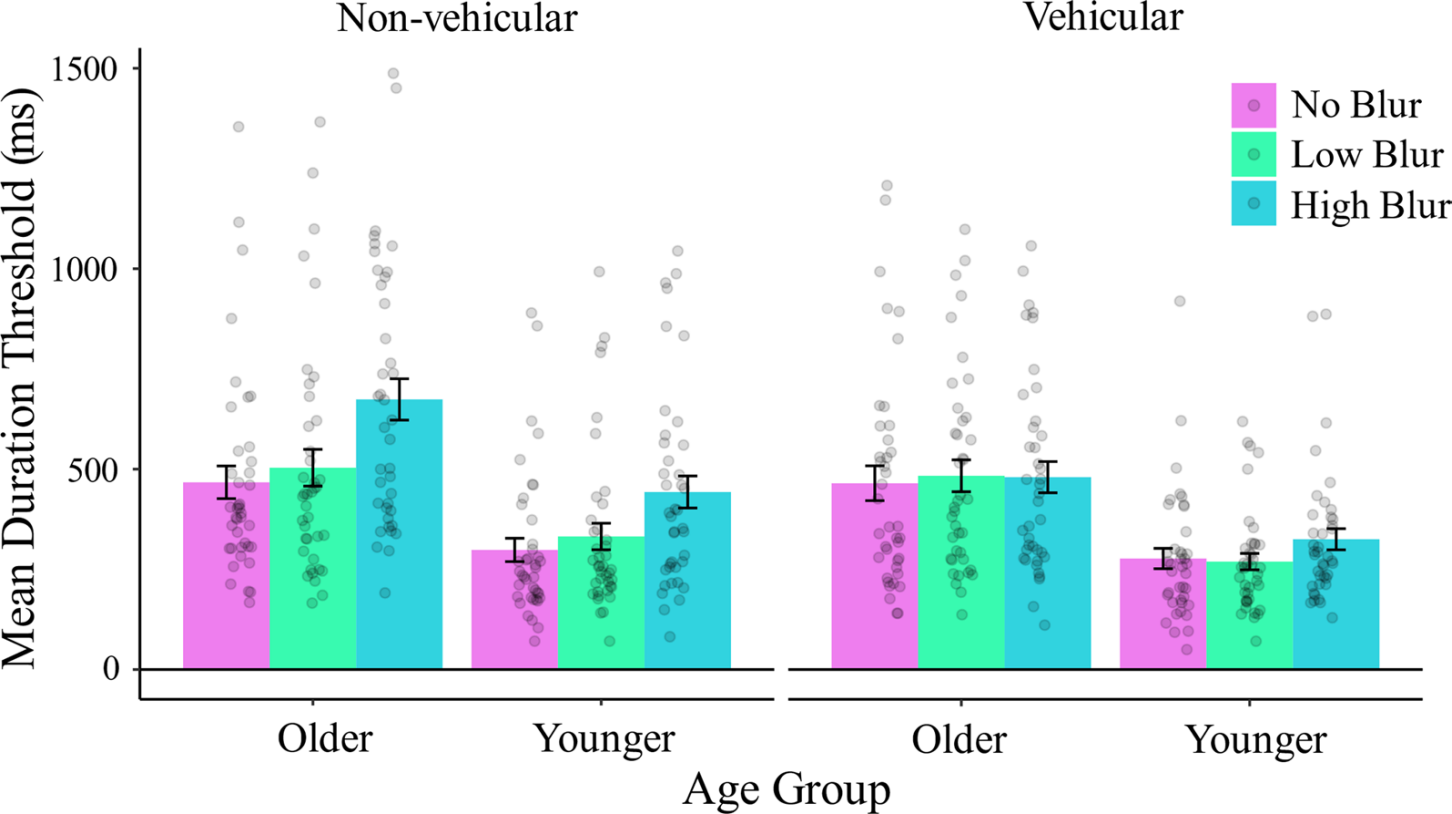
Results, Experiment 2. Bar plots showing mean duration thresholds by hazard type, age group, and blur condition. The magenta bars represent the no blur condition, the green bars represent the low blur condition, and the cyan bars represent the high blur condition. Error bars are ± 1 standard error of the mean, and each point represents one participant. Blur affected non-vehicular hazard thresholds significantly, while vehicular hazards were unaffected by blur. Low blur did not significantly affect duration thresholds

In Experiment 2, we replicated the significant main effect of Age Group, F(1,78) = 24.70, *p* < 0.001, *η*_*p*_^2^ = 0.24, with higher duration thresholds for older (mean = 512 ± 283 ms) compared to younger adults (mean = 324 ± 195 ms) (Fig. 3). As in Experiment 1, there was also a main effect of Blur Level, F(1.80,140.12) = 13.90, *p* < 0.001, *η*_*p*_^2^ = 0.15. Averaged across hazard type, we observed higher duration thresholds for the high blur condition (mean = 480 ± 282 ms) compared to low blur (mean = 397 ± 247 ms) and no blur (mean = 377 ± 240 ms). In addition, we observed a significant main effect of Hazard Type, F(1,78) = 18.34, *p* < 0.001, *η*_*p*_^2^ = 0.19. Collapsed across blur levels, we observed higher duration thresholds for non-vehicular hazards (mean = 453 ± 283 ms) compared to vehicular hazards (mean = 383 ± 230 ms). These main effects are qualified by a significant interaction between Blur Level and Hazard Type, F(1.68,130.71) = 7.61, *p* = 0.001, *η*_*p*_^2^ = 0.09, such that the effect of blur was significant only for non-vehicular hazards. For non-vehicular hazards, pairwise contrasts showed significantly higher thresholds in the high blur compared to no blur condition *t*(78) = 5.09, *p* < 0.001, and higher thresholds in the high blur compared to low blur condition *t*(78) = 3.99, *p* < 0.001. In contrast to our predictions, there was no significant difference in thresholds between the no blur and low blur conditions, *t*(78) = 1.29, *p* = 0.35. No pairwise comparisons were significant for vehicular hazards, *t*(78) < 1.3, *p* > 0.42. No other interactions were significant (all *p* values > 0.27).

## Discussion

Prior work has demonstrated that blur can impact drivers’ ability to control their vehicle and may slow their responses, but these measures reflect a combination of perceptual, decisional and response processes. Here, we isolated the perceptual impact of blur by measuring duration thresholds for road hazard detection—the viewing time required to accurately detect a hazard—and compared this measure between different age groups and hazard types (vehicular and non-vehicular). Across our experiments, we observed a general effect of blur across age groups, wherein both older and younger adults showed elevated duration thresholds when viewing blurred videos. In Experiment 1, which contained a mix of vehicular and non-vehicular hazards, a high level of blur increased thresholds by 78 ms on average. Experiment 2 demonstrated that this effect is likely driven by impaired recognition and understanding of situations with non-vehicular hazards (pedestrians, cyclists, and animals), as the same blur level increased thresholds by 176 ms for non-vehicular hazards (equivalent to a car length at highway speeds), but had minimal effect on vehicular hazard detection. This suggests that there may be mild levels of blur, at which a vehicular hazard would be readily detectable, but a pedestrian would be more challenging to detect. Although duration thresholds for vehicular hazards were stable at the highest levels of blur that we tested, this does not exclude the possibility that comparable levels of blur may have an impact in more naturalistic scenarios; determining the consequences for vehicle operation would require further investigation.

Together, these results highlight the importance of separately assessing effects of blur for specific classes of road hazards. However, we note that, in naturalistic scenarios, many additional factors, such as physical size, movement speed, visual attention, and the potential consequences of a collision can potentially influence hazard perception. It is possible that these factors might contribute to the difference we observed in the impact of blur between non-vehicular and vehicular hazards, as well as detection of these different categories of hazards more broadly. In particular, different potential hazards move at different speeds, and future studies with simulated road scenarios might probe the specific question of speed and time-to-collision beyond what can be achieved with our videos.

In addition, in the world, the time one needs to detect a hazard is, of course, measured from hazard onset, or the earliest deviation of an object from normal behavior. In other words, drivers on the road have the benefit of viewing the road scene leading up to the hazard, whereas participants in our study did not have this context, and viewed brief videos which began after hazard onset. However, a previous study with the same video set (Wolfe et al., 2020) showed that this additional context does not significantly impact duration thresholds (although they may impact on road behavior), as there was no difference in thresholds between conditions in which participants were shown clips that started before versus after hazard onset. Our use of video segments starting after hazard onset, while less representative of on-road scenarios, was intended to reduce variability in threshold estimates, as hazards were clearly visible in the non-blurred versions of the videos.

Most surprisingly, we found no interaction between blur and age in either experiment, suggesting that younger and older participants were similarly affected by blur. These results agree broadly with those of Lee et al. (2016), who found a similar universal impact of blur on HPT performance, wherein both older and younger adults had delayed reaction times to hazards in videos of road scenes, with reaction time increasing approximately 300–400 ms. While the stimulus sets were not identical, the most comparable condition in our experiment (High blur; Exp 1) elevated duration thresholds by 78 ms, suggesting that blur may impact multiple stages of the response process.

Our result showing comparable effects of blur in older and younger adults contrasts with work showing that older adults may be less resistant to the effects of blur in other settings (e.g., Bartel & Kline, 2002; Wolfe et al., 2016) such as reading. However, understanding a dynamic road scene is very different from reading, which is dependent on high spatial frequency information to a far greater degree than our stimuli, and may simply be more vulnerable to blur.

A common thread across these studies, however, is that both studies blurred their respective stimuli computationally, which does not reflect the true nature of blur outside the laboratory. Real-world blur produced by optical defocus is distance dependent, and future work could blur stimuli optically, rather than computationally, to determine whether these results hold under more realistic conditions. Furthermore, additional work is also needed to examine how decrements in contrast sensitivity would impact the detection of vehicular and nonvehicular road hazards. As small amounts of blur produce decrements in contrast sensitivity at a range of medium-to-high spatial frequencies (Marmor & Gawande, 1988), this could contribute to impaired hazard detection in our study. However, our blur conditions only simulate the vision impairments that would be associated with uncorrected refractive error in normally-sighted observers. Importantly, any decrements in contrast sensitivity would be more severe for individuals with vision loss due to ocular disease, and could have a much larger impact on hazard detection.

Together, our results suggest that while blur similarly impacts different age groups of drivers, it has a specific and dangerous impact on vulnerable road users (pedestrians and cyclists). Understanding that we share the road, and the particular vulnerability of our fellow road users, may point to a need for multifaceted interventions to improve road safety. These may include additional vision screening, but also engineering improvements in the vehicle to alert drivers to their fellow road users.

### Supplementary Information

Supplementary Material 1.

## Data Availability

Both experiments were preregistered on the Open Science Framework (Experiment 1, https://osf.io/czk9g Experiment 2, https://osf.io/3zp7e), and all stimuli, code, and data are deposited at https://osf.io/qtd6p/

## Footnotes

1 We note that this was not a pre-registered criterion, but was added following inspection of the demographic data, which revealed mismatches between participants’ self-reported age and the information from their profile.
