# Supplementary material for: Seeing the truck, but missing the cyclist: effects of blur on duration thresholds for road hazard detection
Source: Cogn Res Princ Implic. 2024 May 20;9:32. doi: 10.1186/s41235-024-00557-7 (PMC11106223; doi:10.1186/s41235-024-00557-7)
Supplement: Supplementary file 1 — Supplementary Material 1. [file 41235_2024_557_MOESM1_ESM.pdf]

**Supplemental Materials**
**Title: Seeing the truck, but missing the cyclist: Effects of blur on duration thresholds for road hazard detection**
**Authors:** Silvia Guidi, Anna Kosovicheva, Benjamin Wolfe

**Supplemental Note 1: Replication of Wolfe, B., Seppelt, B., Mehler, B., Reimer, B., & Rosenholtz, R. (2020). Rapid holistic perception and evasion of road hazards. *Journal of Experimental Psychology. General,* 149(3), 490–500.**

Prior to data collection in the present study, we conducted a separate online experiment to verify the reliability and stimulus timing of online experiments by replicating a previous in-person laboratory study (Wolfe et al., 2020) that measured duration thresholds for the detection of road hazards in dashboard camera videos. A total of 46 participants completed the study through Prolific, with the same eligibility and exclusion criteria as Experiments 1 and 2. A total of six participants were removed from the analysis based on the same exclusion criteria outlined in the Analysis section. The final sample consisted of 21 older adults (ages 55-70; mean age 61.1; SD = 4.8) and 19 younger adults (ages 20-35; mean age; 25.4; SD = 3.6). Procedures were identical to those described in Experiment 1, except none of the videos were blurred. Duration thresholds were estimated from a single staircase consisting of 100 trials. Data are plotted side-by-side with data from Wolfe et al. 2020 (response-locked condition), which used the same video set in an in-person experiment with a separate group of normally-sighted observers with comparable ages (M = 25.8; SD = 3.7 for younger adults; M=63.7 SD: 3.8 for older adults). The task and procedures of the online study attempted to match the in-person experiment as much as possible.

Figure S1 shows the results from each experiment. Thresholds were compared using a 2 x 2 ANOVA, with age group (younger vs older) and experiment (in-person vs online) as between-subjects factors. There was a significant main effect of age group; on average, duration

thresholds were higher for older adults compared to younger adults (406 vs. 224 ms; F(1,75) = 20.97, $p < 0.001$, $\eta_p^2 = 0.22$). There was no significant main effect of experiment ($F(1,75) = .03$, $p = 0.87$), and no interaction between age group and experiment, $F(1,75) = .001$, $p = 0.98$. The close correspondence between thresholds measured in-person and online (403 vs. 408 ms for older adults; 220 vs. 228 ms for younger adults) points to the reliability of duration threshold measurement for road hazard detection in online settings.

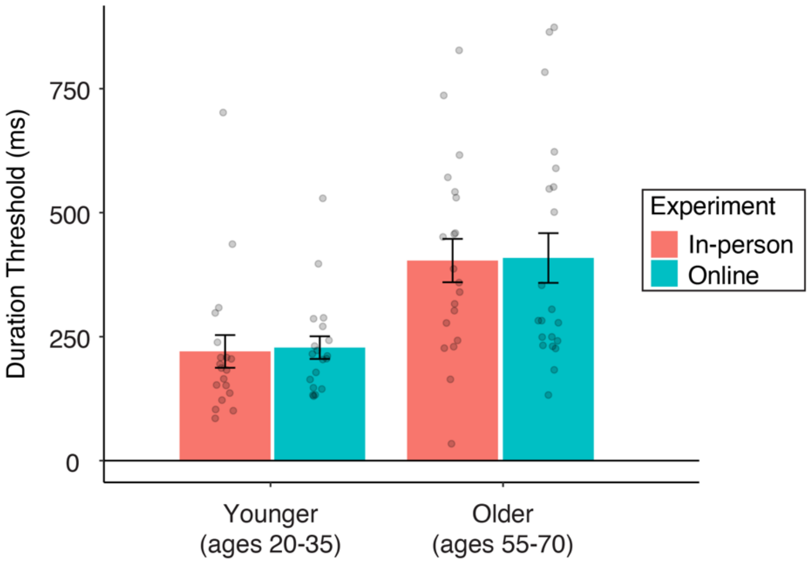


**Figure S1.** Results for a comparable in-person study (Wolfe et al., 2020; response-locked condition; coral) plotted together with data from an online experiment (teal) with an identical procedure to the no-blur condition in Experiment 1. Error bars represent ±1 standard error of mean and each point represents one participant's threshold.

**Supplemental Note 2: Re-analysis of Experiments 1 and 2 based on average of staircase reversals**

For each experiment, we replicated the main analysis using duration thresholds obtained by averaging the reversal points of each staircase. In this analysis, no participants were excluded, and we obtained similar results, indicating that excluding participants with poor psychometric fits did not affect our results. In Experiment 1, There was a significant main effect of Age

Group, F(1,81) = 14.14, $p$<0.001, $\eta_p^2$ = 0.15. We also found a significant main effect of Blur, F(1,81) = 14.65, $p$<0.001, $\eta_p^2$ = 0.15, with higher duration thresholds in the high blur condition. As in the main analysis Age Group did not significantly interact with Blur Level, F(1,81) = 0.00, $p$ = 0.99, $\eta_p^2$ = <0.001

In Experiment 2, we observed a main effect of Age Group, F(1,99) = 29.11, $p$ < 0.001, $\eta_p^2$ = 0.23, as well as a main effect of Blur Level, F(1.90,187.92) = 16.62, $p$ < 0.001, $\eta_p^2$ = 0.14 and Hazard Type, F(1,99) = 12.36, $p$ < 0.001, $\eta_p^2$ = 0.11.We found a significant interaction between Blur Level and Hazard Type, F(1.88,185.88) = 9.68, $p$ < 0.001, $\eta_p^2$ = 0.089. As in the previous analysis, none of the other interactions were significant (p-values > 0.17).
